# Experimental Coma

**Published:** 1911-10

**Authors:** 


### Experimental Coma

Ehrmann and Esser, *Zeit. für Klin. Med.,* 1911, cite the production of typic coma in the dog, by von Marx, by intraperitoneal injection of sodium butyrate. The authors confirm the general principle by administration of butyric acid by mouth to rabbits; 3—5 grams per kilo. producing the ordinary clinical symptoms of diabetic coma.

